# Human Pegivirus in Patients with Encephalitis of Unclear Etiology, Poland

**DOI:** 10.3201/eid2410.180161

**Published:** 2018-10

**Authors:** Iwona Bukowska-Ośko, Karol Perlejewski, Agnieszka Pawełczyk, Małgorzata Rydzanicz, Agnieszka Pollak, Marta Popiel, Kamila Caraballo Cortés, Marcin Paciorek, Andrzej Horban, Tomasz Dzieciątkowski, Marek Radkowski, Tomasz Laskus

**Affiliations:** Medical University of Warsaw, Warsaw, Poland (I. Bukowska-Ośko, K. Perlejewski, A. Pawełczyk, M. Rydzanicz, M. Popiel, K. Caraballo Cortés, M. Paciorek, A. Horban, T. Dzieciątkowski, M. Radkowski, T. Laskus);; Institute of Physiology and Pathology of Hearing, Kajetany, Poland (A. Pollak)

**Keywords:** human pegivirus, pegivirus, GB virus C, hepatitis, encephalitis, compartmentalization, viruses, Poland

## Abstract

Human pegivirus (HPgV), previously called hepatitis G virus or GB virus C, is a lymphotropic virus with undefined pathology. Because many viruses from the family Flaviviridae, to which HPgV belongs, are neurotropic, we studied whether HPgV could infect the central nervous system. We tested serum and cerebrospinal fluid samples from 96 patients with a diagnosis of encephalitis for a variety of pathogens by molecular methods and serology; we also tested for autoantibodies against neuronal antigens. We found HPgV in serum and cerebrospinal fluid from 3 patients who had encephalitis of unclear origin; that is, all the markers that had been tested were negative. Single-strand confirmation polymorphism and next-generation sequencing analysis revealed differences between the serum and cerebrospinal fluid–derived viral sequences, which is compatible with the presence of a separate HPgV compartment in the central nervous system. It is unclear whether HPgV was directly responsible for encephalitis in these patients.

## Abstract 2

Sequence analysis of human pegivirus from 3 patients indicates that the central nervous system constitutes a separate viral compartment from serum.

Human pegivirus (HPgV) was originally described as a hepatitis virus by 2 independent groups of researchers and called GB virus C and hepatitis G virus (*1*,*2*). Whereas the infection was found to be common in patients with forms of chronic hepatitis, and particularly prevalent in patients with chronic hepatitis C infection, it is not associated with liver injury in the absence of concomitant infection with hepatitis C virus (HCV) or hepatitis B virus (HBV). Furthermore, the liver is not the primary replication site for this virus (*3*,*4*). This virus was recently renamed as pegivirus and assigned to a new genus (*Pegivirus*) within the family Flaviviridae (*5*). 

Infection with HPgV is common worldwide; ≈5% of healthy blood donors in industrialized countries are viremic, whereas in some developing countries the prevalence of viremia among blood donors is ≈20% (*6*). There is evidence that HPgV is transmitted parenterally, sexually, and also vertically from mother to child (*7*). However, the high proportion of HPgV infection in apparently healthy blood donors and the general population suggests existence of nonparenteral routes. The reasons for the high prevalence of infection in developing countries are not entirely clear but could be related to overall poor hygienic conditions, as well as to the time of exposure. In sub-Saharan Africa, where HPgV is particularly common, this virus is transmitted mainly during childhood, which may facilitate the establishment of chronic infection (*7*). Because no association between HPgV and disease has been consistently identified, blood donors are not routinely screened for the virus.

Interest in the HPgV infection was revived when several studies identified its beneficial effect on the survival of HIV-infected persons (*8*,*9*); anti-HIV replication effects of HPgV were confirmed in vitro (*8*). Several in vivo and in vitro studies suggest that HPgV may directly interfere with HIV replication and affect host cell factors necessary for the HIV life cycle; specific mechanisms include modulation of cytokine and chemokine release and receptor expressions and lowering of T-cell activation and proliferation (*9*). However, infection with HPgV may not be totally benign; some studies found an association between infection and non-Hodgkin lymphoma (*10*,*11*), which could be the result of lowered immune activation.

Many viruses from the family Flaviviridae, most prominently arthropodborne viruses (arboviruses) such as West Nile virus (WNV) and tick-borne encephalitis virus (TBEV), are neurotropic and a prominent cause of encephalitis in Europe and North America (*12*). These factors raise the question whether HPgV could be neurotropic and whether it could be an etiologic agent in neuroinfections. Of note, despite substantial progress in diagnostics, the etiology of encephalitis remains unclear in 40%–80% of patients (*13*,*14*). A plethora of pathogens may cause encephalitis; many of these pathogens are rare and thus testing is not performed to identify them, and others have not yet been identified. 

Three recent case reports described HPgV RNA in the human central nervous system (CNS), demonstrating that the virus can be present in the brain under certain circumstances (*15*–*17*). In the first study, viral sequences were detected postmortem in brain tissue from a patient with multiple sclerosis, not encephalitis (*15*). In the second study, the presence of HPgV might have been related to a severely compromised blood–brain barrier; the patient was HIV-positive and had cerebral toxoplasmosis and fungal encephalitis (*16*). Although the full-length virus was recovered from the patient’s brain tissue, it is unclear which cells harbored the virus and it was possible that the actual source was blood. Furthermore, the association of HPgV with multiple sclerosis could not be established because the study was limited to a single case. In the third study, HPgV was detected in serum and CSF of a patient with a severe form of encephalitis of unclear origin (*17*). Of these 3 studies, none included comparison of serum- and CNS-derived virus. We conducted a study of 96 consecutive patients with diagnosis of encephalitis (*18*) in Poland during 2012–2015 to determine whether HPgV could be found in the CNS. 

## Materials and Methods

### Patients and Routine Diagnostics

We prospectively enrolled patients with encephalitis at the Warsaw Hospital for Infectious Diseases (Warsaw, Poland) from June 2012 through July 2015. The details of this study were published previously (*18*). We defined encephalitis as an acute-onset illness with altered mental status, decreased level of consciousness, seizures, or focal neurologic signs, together with >1 abnormality of the CSF (leukocyte count >4 cells/mm^2^ or protein level >40 mg/dL). We obtained written informed consent from all patients or from close relatives of patients unable to give consent due to their condition. The Internal Review Board of the Medical University of Warsaw approved the study.

We collected CSF and serum samples from patients at admission (5–7 days after symptom onset) and kept them frozen at −80°C until analysis. We tested the samples from all 96 patients for the presence of 5′ untranslated region (UTR) HPgV RNA. We performed real-time quantitative PCR (qPCR) or real-time quantitative reverse transcription PCR (qRT-PCR) to detect human herpesvirus (HHV) 1 and 2, varicella zoster virus (VZV), cytomegalovirus (CMV), HHV-6, enteroviruses (coxsackievirus A9, A16, B2, B3, B4, B5; echovirus 5, 6, 9, 11, 18, 30; and enterovirus 71), TBEV, WNV, and human adenovirus (HAdV) in CSF samples. We used commercial serologic tests to test CSF and paired serum samples for HHV-1, HHV-2, VZV, TBEV, and WNV as described (*18*). We detected autoantibodies against neuronal surface antigens using the Autoimmune Encephalitis Mosaic 6 assay (Euroimmun AG, Luebeck, Germany).

### HPgV 5′ UTR and E2 Amplification

We extracted total RNA with TRIzol LS (ThermoFisher Scientific, Waltham, MA, USA) from 400 µL of CSF or serum and suspended RNA in 20 µL of water, 5 µL of which was subsequently used for each amplification reaction. We amplified the HPgV 5′ UTR by nested reverse transcription PCR (RT-PCR) as described previously (*19*), resulting in 421 bp-length product; we amplified the E2 region following the RT-PCR protocol published by Smith et al. (*20*). The final product was 422 bp in length.

### Single-Strand Conformation Polymorphism

We subjected the amplified PCR products to single-strand conformational polymorphism analysis, as described previously (*21*). In brief, we purified HPgV 5′ UTR and HPgV-E2 PCR products by using the Wizard PCR Preps DNA Purification System (Promega, Madison, WI, USA). We then subjected the products to thermal denaturation, ran them on nondenaturing 1% polyacrylamide gels at 400V in 25°C, fixed them with acetic acid, and stained them with silver stain.

### Next-Generation Sequencing

We reamplified RT-PCR products with primers specifically designed for the Illumina MiSeq platform (Illumina, San Diego, CA, USA). Each primer contained the following: sequences complementary to the adapters on a flow cell; an 8-nt index sequence; sequences corresponding to the Illumina sequencing primers; and sequence-specific nested primers for the 5′ UTR and E2 region. Amplification of the 5′ UTR region included initial denaturation at 94°C for 5 min, 20 cycles of 94°C for 1 min, 58°C for 1 min, 72°C for 1 min, and final elongation at 72°C for 10 min. HPgV E2 amplification included denaturation at 94°C for 5 min followed by 20 cycles of 94°C for 18 s, 55°C for 20 s, 72°C for 90 s, and 1 cycle at 72°C for 10 min. We trimmed the libraries by using the LabChip XT apparatus (PerkinElmer, Waltham, Massachusetts, USA) with the DNA 300 Assay Kit (PerkinElmer); the range of fraction collection was 370–430 bp for 5′ UTR and 460–530 bp for E2.

We assessed the quality and average length of next-generation sequencing libraries by using Bioanalyzer (Agilent Technologies, Santa Clara, CA, USA). We equimolarily pooled the indexed samples and sequenced them on Illumina MiSeq with 301 bp-end reads according to the manufacturer’s protocol.

### Data Analysis

We trimmed raw reads using cutadapt version 1.2.1 (https://github.com/marcelm/cutadapt/); (*22*), then used FASTX-Toolkit (http://hannonlab.cshl.edu/fastx_toolkit/index.html) for additional processing. We removed all Phred quality score reads <20 using FASTQ/A Artifacts Filter and preprocessed the remaining reads (grouping, counting, and frequency arrangement) using R scripts (*23*). To diminish the contribution of false positive variants to genetic diversity, we applied the experimentally established sequencing error cutoff of 1.22%. Finally, we aligned remaining sequences and generated phylogenic trees with ClustalX version 2.0 (http://www.clustal.org/clustal2/) (*24*). We assessed nucleotide diversity per site and the number of substitutions with respect to the dominant serum sequence in each patient using DnaSP version 6.11.06 (*25*). We predicted the RNA secondary structures of the 5′ UTR using Mfold version 3.2 (http://unafold.rna.albany.edu/?q=mfold) (*26*), and searched for putative B cell epitopes within the E2 region using BepiPred-2.0 (http://www.cbs.dtu.dk/services/BepiPred/) (*27*).

## Results

Four patients were positive for 5′ UTR HPgV RNA in serum, and 3 of these patients were also positive in CSF. We analyzed the samples from those 3 patients; their samples were collected at admission, which was 5–7 days after symptom onset. We diagnosed encephalitis of unclear origin for all 3 patients because they were negative for all the pathogens tested (Table 1). The small number of HPgV-infected patients did not allow for statistical analysis, but these patients were not strikingly different from other encephalitis patients. We initially suspected 1 patient, who had a severe illness with prolonged hospitalization, of having HHV infection, but tests did not confirm HHV. All 3 patients recovered without any neurologic sequelae.

**Table 1:** Comparison of clinical characteristics of 3 encephalitis patients infected with human pegivirus compared with patients with other forms of encephalitis, Poland, 2012–2015*

Characteristic	Encephalitis patients infected with pegivirus				Other encephalitis patients	
	Patient 1	Patient 2	Patient 3		Infectious cause identified, n = 41†	Unknown cause or autoimmune illness, n = 52‡
Male sex	No	No	Yes		30 (73)	23 (44)
Median age, y (range)	55	28	20		38 (19–85)	38 (20–82)
Pharmacological immunosuppression present	0	0	0		2 (5)	3 (6)
HIV positive	0	0	0		0	2 (4)
Cancer	0	0	0		6 (15)	1 (2)
Median length of hospital stay, d (range)	41	30	8		12 (5–97)	11.5 (6–79)
Fever >38°C	Yes	Yes	No		26 (63)	18 (35)
Headache	Yes	Yes	No		22 (54)	25 (48)
Altered mental status	Yes	Yes	No		36 (88)	47 (85)
Focal neurologic signs	No	No	Yes		9 (22)	10 (20)
Seizures	No	No	Yes		12 (29)	16 (31)
Stiff neck	No	No	No		7 (17)	12 (23)
CSF analysis						
Median leukocyte count, cells/mm^2^ (range)	3	4	3		41 (1–1225)	18 (1–362)
Median protein level, g/L (range)	0.68	0.26	1.63		0.57 (0.16–3.21)	0.56 (0.11–3.33)
Death	0	0	0		1 (2.4)	0

*Values are no. (%) unless otherwise indicated. CSF, cerebrospinal fluid †Identified infections were human herpesvirus 1 (n = 22), enterovirus (n = 6), varicella zoster virus (n = 5), tick-borne encephalitis virus (n = 6), and cytomegalovirus (n = 2). ‡All markers of viral infection were negative, but 5 patients had antibodies against N-methyl-D-aspartate receptor and a diagnosis of autoimmune encephalitis.

**Figure 1:**
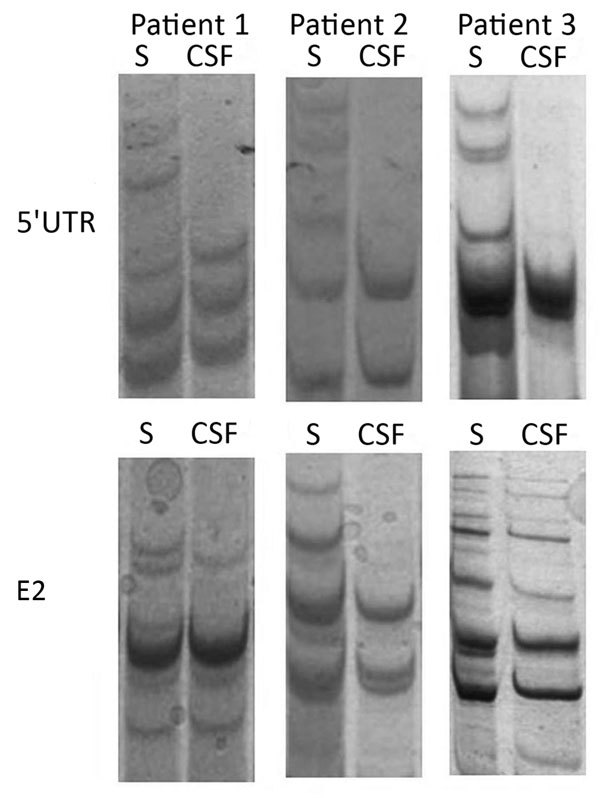
Single-strand conformation polymorphism analysis of 5′ UTR and E2 region human pegivirus amplicons from 3 patients with encephalitis of unclear origin, Poland, 2012–2015. CSF, cerebrospinal fluid; S, serum; UTR, untranslated region.

We compared serum- and CSF-derived 5′ UTR and E2 amplicons from the 3 patients by single-strand conformational polymorphism analysis (Figure 1). Because this analysis suggested the presence of differences between the serum- and CSF-derived viral sequences in individual patients, we subjected all amplicons to next-generation sequencing. After filtering, the mean number of reads per sample was 70,759 (range 2,706–183,046) (Table 2).

**Table 2:** 5 Human pegivirus variants in serum and cerebrospinal fluid in 3 patients with encephalitis of unknown origin, Poland, 2012–2015*

RNA region	Patient 1			Patient 2			Patient 3	
	Serum	CSF		Serum	CSF		Serum	CSF
5′ untranslated region								
No. reads before error cutoff	124,987	110,331		108,002	183,046		101,240	145,411
No. reads after error cutoff	61,783	57,668		49,075	109,061		55,328	75,998
No. nucleotide variants†	3	7		3	3		2	3
No. unique nucleotide variants in CSF†	–	5 (29.32)		–	1 (2.28)		–	1 (2.49)
No. nucleotide substitutions	2	7		2	2		1	2
Nucleotide diversity per site	0.004	0.007		0.004	0.004		0.003	0.004
E2 region								
No. reads before error cutoff	70,460	38,025		77,656	76,918		2,706	82,738
No. reads after error cutoff	26,720	20,619		26,558	42,609		453	34,887
No. nucleotide variants†	8	4		8	9		4	7
No. unique nucleotide variants in CSF†	–	0		–	5 (41.78)		–	3 (27.28)
No. nucleotide substitutions	4	2		5	5		2	3
Nucleotide diversity per site	0.007	0.004		0.006	0.007		0.004	0.005
No. amino acid variants†	2	2		2	5		2	4
No. unique amino acid variants in CSF†	–	0		–	3 (27.28)		–	2 (27.28)

*Numbers in parentheses are percentages. CSF, cerebrospinal fluid †After applying the 1.22% sequencing error cutoff.

**Figure 2:**
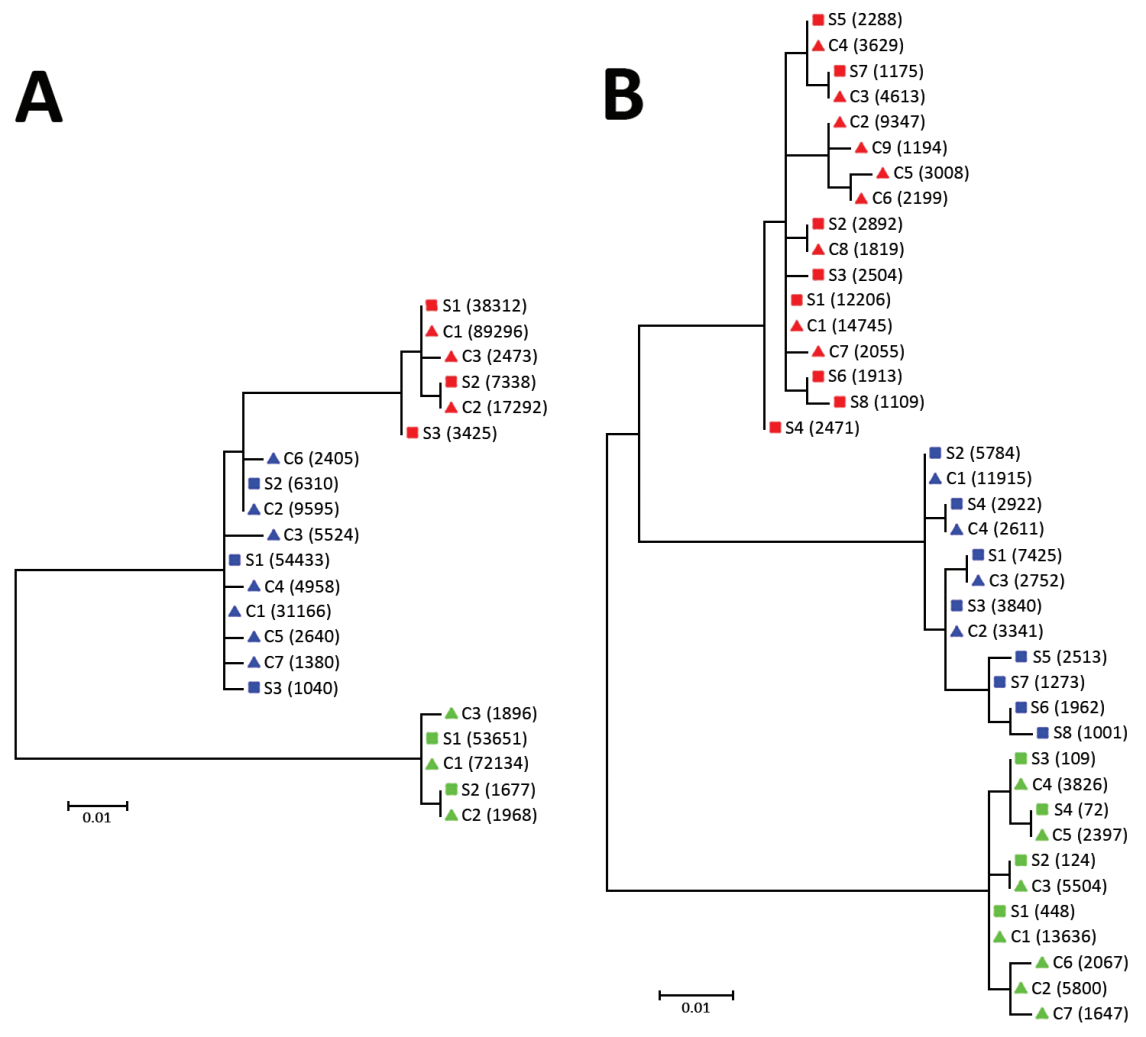
Phylogenetic analysis of A) 5′ UTR and B) E2 region sequences of human pegivirus from 3 patients with encephalitis of unclear origin, Poland, 2012–2015. Phylogenic trees were generated using ClustalX version 2.0 (http://www.clustal.org/clustal2/). Viral variant frequencies follow haplotype number. Red indicates patient 1; blue, patient 2; green, patient 3. Scale bars indicate number of nucleotide substitutions per site. C, cerebrospinal fluid; S, serum; UTR, untranslated region.

**Figure 3:**
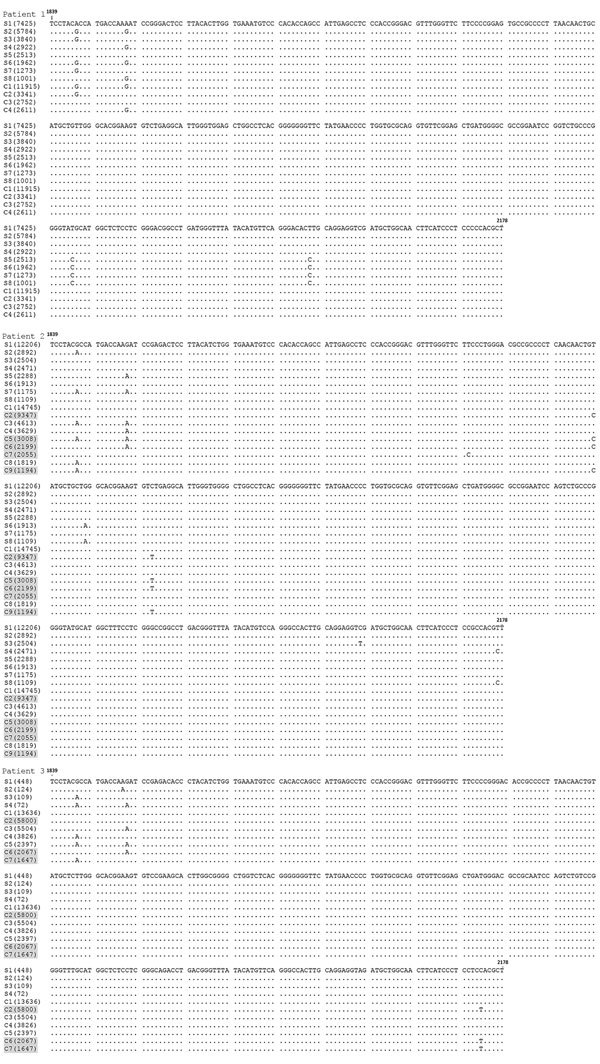
Comparison of E2 region human pegivirus sequences amplified from serum and cerebrospinal fluid from 3 patients with encephalitis of unclear origin, Poland, 2012–2015. Numbers in parentheses represent the number of reads representing a given sequence. Shading indicates sequences unique to cerebrospinal fluid. Nucleotide numbering follows the reference strain published by Linnen et al (*2*) (GenBank accession no. NC_001710.1). C, cerebrospinal fluid; S, serum.

**Figure 4:**
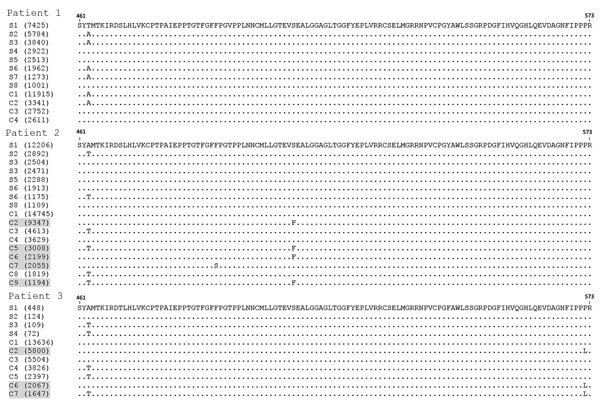
Comparison of amino acid composition of E2 region human pegivirus sequence variants amplified from serum and cerebrospinal fluid from 3 patients with encephalitis of unclear origin, Poland, 2012–2015. Numbers in parentheses represent the number of reads representing a given sequence. Shading indicates sequences unique to cerebrospinal fluid. C, cerebrospinal fluid; S, serum.

When we compared 5′ UTR and E2 sequences phylogenetically, we found that serum- and CSF-derived sequences clustered together in individual patients; no sequence was found in multiple patients (Figure 2). Of note, we found several variants to be unique in the CSF compartment. These sequences comprised 2.28%–29.32% of all variants for 5′ UTR and 0%–41.78% of all variants for E2 (Table 2; Figure 3). Unique CSF-derived sequences were also present when we analyzed the E2 region on the amino acid level (Figure 4). The changes were serine to phenylalanine at aa position 508 in patient 2 and proline to leucine at position 572 in patient 3. Both changes were within the predicted B cell epitopes (aa 506–522 and 559–572).

We analyzed all 5′ UTR sequence variants to determine the predicted stability of their secondary RNA structure. We deemed the effect of variations minor because most were localized in the nonbasepaired parts (data not shown), and free energies of the hypothetical secondary structures were only occasionally and mildly affected (Technical Appendix Figure) (*28*).

## Discussion

We detected HPgV sequences in CSF from 3/96 patients with encephalitis. We classified all 3 cases as encephalitis of unknown origin because they were negative for serologic and molecular markers of common CNS pathogens. Furthermore, we demonstrated that these viral sequences differed from those circulating in serum. The presence of viral RNA in CSF could be due to a compromised blood–brain barrier, which was possible in these patients with encephalitis. However, the presence of differences in circulating and CSF-derived sequences is more compatible with the existence of separate viral compartments and thus independent replication. Similar compartmentalization in which distinct blood and CNS viral populations indicate separately evolving populations has been described for other viruses, most prominently for HIV (*29*) but also for HCV (*30*,*31*) and human BK polyomavirus (*32*). 

It is unclear how HPgV could access the CNS. Although initially HPgV was thought to be a hepatotropic virus, the viral negative strand, which is the putative replicative intermediate of the virus, was not detected in liver tissue (*3*); however, it was found in bone marrow and spleen (*33*,*34*). The virus is now considered lymphotropic because it can be detected at a low level in multiple lineages of peripheral leukocytes (*35*,*36*), and it has been speculated that the primary target could be a progenitor hematopoietic stem cell. The route for CNS access could be through infected leukocytes; all basic groups (T cells, B cells, macrophage/monocytes, and NK cells) have the ability to enter the brain under certain conditions (*37*). Certain monocyte family members are constantly replaced as part of normal physiology (*38*,*39*), while the entry of T cells and B cells depends largely on their activation state (*40*,*41*). Such a phenomenon related to trafficking of infected leukocytes through the blood–brain barrier has been long postulated for HIV-1 neuroinfection (*42*).

Whether HPgV was the causative factor of encephalitis in the patients we describe is not clear. Viral pathogens are typically present only transiently in CSF and this window could be easily missed, particularly when the spinal tap is done too late in the course of illness (*43*). If HPgV encephalitis exists, it could be a rare phenomenon. In a previous study of 17 encephalitis and aseptic meningitis cases of unknown cause, no CSF sample was positive (*44*). However, even infections with well-known neurotropic agents from the Flaviviridae family, such as WNV or TBEV, are usually subclinical or asymptomatic; clinical signs and symptoms develop only in 5%–30% of cases (*45*,*46*). Of note, in 2 of the cases we describe, the encephalitis was mild, and all 3 patients recovered without any neurologic sequelae. Obviously, the mere presence of a pathogen in the CNS in patients with encephalitis does not prove causality; for example, HCV sequences are commonly detected in brain and CSF of infected patients without any accompanying evidence of encephalitis (*29*). In our study, we detected HPgV sequences in CSF only in patients without an obvious cause of encephalitis and in none of the patients in whom a known pathogen was identified.

The identified 5′ UTR and E2 region sequence differences between CSF and serum compartments could have biological meaning. The 5′ UTR contains an internal ribosomal entry site that allows cap-independent viral translation (*28*). Such structures were identified within the 5′ UTR of the picornaviruses and were shown to interact with cellular proteins, thus affecting the host range of individual viruses (*47*). Research has also shown that, for HCV, translation efficiencies of brain-derived internal ribosomal entry site variants are generally lower than those found in serum, which could be a viral strategy favoring latency in the CNS (*31*). Taking this into consideration, we speculated that at least some of the 5′ UTR changes in the patients we report represent tissue-specific adjustment. Viral adaptive changes could be relatively small and yet make a huge difference; for example, it has been demonstrated for lymphocytic choriomeningitis virus in mice that variants differing by a single amino acid substitution are competitively selected either by the liver and spleen or by neurons (*48*).

On the amino acid level, we saw 2 unique E2 region changes in CSF variants compared with serum: in patient 2, serine was changed to phenylalanine at aa position 508, and in patient 3, proline was changed to leucine at position 572. Both were within regions predicted to contain B-cell epitopes, thus suggesting that they were the effect of immune pressure. Furthermore, the change in patient 3 was located in the region of E2 that was experimentally shown to contain a strong antigenic site and likely to be involved in cell binding or fusion (*49*).

RNA viruses in particular are characterized by a high degree of genetic heterogeneity; probably because the lack of proofreading 3′–5′ exonuclease activity in viral RNA polymerases causes low fidelity. As a result, viruses circulate in the infected host as a population of closely related but nonidentical genomes, referred to as quasispecies (*50*). It is unclear whether the observed high HPgV variability developed in the patients we describe de novo after infection or if most or all variants were transmitted from the infecting host; both mechanisms could occur together. However, because viral transmission is typically accompanied by narrowing of the quasispecies spectrum (known as the bottleneck phenomenon), some extent of postinfection evolution is highly likely.

In summary, we detected HPgV sequences in the CSF of 3 patients with encephalitis of unclear origin, and these sequences from CSF differed from those circulating in serum. These findings are compatible with the presence of a separate viral compartment in the CNS. Determining if the pegivirus was responsible for encephalitis or if it was present along with another cause of encephalitis will require further research, including histopathological analysis.

Technical Appendix Additional information about human pegivirus in patients with encephalitis, Poland.

## References

[1] Simons JN, Leary TP, Dawson GJ, Pilot-Matias TJ, Muerhoff AS, Schlauder GG, et al. Isolation of novel virus-like sequences associated with human hepatitis. Nat Med. 1995;1:564–9. 7585124 10.1038/nm0695-564

[2] Linnen J, Wages J Jr, Zhang-Keck ZY, Fry KE, Krawczynski KZ, Alter H, et al. Molecular cloning and disease association of hepatitis G virus: a transfusion-transmissible agent. Science. 1996;271:505–8. 8560265 10.1126/science.271.5248.505

[3] Laskus T, Radkowski M, Wang LF, Vargas H, Rakela J. Lack of evidence for hepatitis G virus replication in the livers of patients coinfected with hepatitis C and G viruses. J Virol. 1997;71:7804–6. 9311866 10.1128/jvi.71.10.7804-7806.1997 PMC192133

[4] Pessoa MG, Terrault NA, Detmer J, Kolberg J, Collins M, Hassoba HM, et al. Quantitation of hepatitis G and C viruses in the liver: evidence that hepatitis G virus is not hepatotropic. Hepatology. 1998;27:877–80. 10.1002/hep.510270335 9500722

[5] Adams MJ, King AM, Carstens EB. Ratification vote on taxonomic proposals to the International Committee on Taxonomy of Viruses (2013). Arch Virol. 2013;158:2023–30. 23580178 10.1007/s00705-013-1688-5

[6] Mohr EL, Stapleton JT. GB virus type C interactions with HIV: the role of envelope glycoproteins. J Viral Hepat. 2009;16:757–68. 19758271 10.1111/j.1365-2893.2009.01194.x PMC3543829

[7] Mphahlele MJ, Lau GK, Carman WF. HGV: the identification, biology and prevalence of an orphan virus. Liver. 1998;18:143–55. 9716223 10.1111/j.1600-0676.1998.tb00142.x

[8] Xiang J, Wünschmann S, Diekema DJ, Klinzman D, Patrick KD, George SL, et al. Effect of coinfection with GB virus C on survival among patients with HIV infection. N Engl J Med. 2001;345:707–14. 11547739 10.1056/NEJMoa003364

[9] Bhattarai N, Stapleton JT. GB virus C: the good boy virus? Trends Microbiol. 2012;20:124–30. 22325031 10.1016/j.tim.2012.01.004 PMC3477489

[10] Nakamura S, Takagi T, Matsuda T. Hepatitis G virus RNA in patients with B-cell non-Hodgkin’s lymphoma. Br J Haematol. 1997;98:1051–2. 9326213

[11] Krajden M, Yu A, Braybrook H, Lai AS, Mak A, Chow R, et al. GBV-C/hepatitis G virus infection and non-Hodgkin lymphoma: a case control study. Int J Cancer. 2010;126:2885–92. 19904755 10.1002/ijc.25035

[12] Leyssen P, De Clercq E, Neyts J. Perspectives for the treatment of infections with Flaviviridae. Clin Microbiol Rev. 2000;13:67–82. 10627492 10.1128/cmr.13.1.67-82.2000 PMC88934

[13] Granerod J, Crowcroft NS. The epidemiology of acute encephalitis. Neuropsychol Rehabil. 2007;17:406–28. 17676528 10.1080/09602010600989620

[14] Granerod J, Tam CC, Crowcroft NS, Davies NW, Borchert M, Thomas SL. Challenge of the unknown. A systematic review of acute encephalitis in non-outbreak situations. Neurology. 2010;75:924–32. 20820004 10.1212/WNL.0b013e3181f11d65

[15] Kriesel JD, Hobbs MR, Jones BB, Milash B, Nagra RM, Fischer KF. Deep sequencing for the detection of virus-like sequences in the brains of patients with multiple sclerosis: detection of GBV-C in human brain. PLoS One. 2012;7:e31886. 22412845 10.1371/journal.pone.0031886 PMC3297595

[16] Liu Z, Zhang Y, Wei F, Xu M, Mou D, Zhang T, et al. Detection of GB virus C genomic sequence in the cerebrospinal fluid of a HIV-infected patient in China: a case report and literature review. Epidemiol Infect. 2016;144:106–12. 26081197 10.1017/S0950268815001326 PMC9507311

[17] Fridholm H, Østergaard Sørensen L, Rosenstierne MW, Nielsen H, Sellebjerg F, Bengård Andersen Å, et al. Human pegivirus detected in a patient with severe encephalitis using a metagenomic pan-virus array. J Clin Virol. 2016;77:5–8. 26872326 10.1016/j.jcv.2016.01.013 PMC7106502

[18] Popiel M, Perlejewski K, Bednarska A, Dzieciątkowski T, Paciorek M, Lipowski D, et al. Viral etiologies in adult patients with encephalitis in Poland: A prospective single center study. PLoS One. 2017;12:e0178481. 28570620 10.1371/journal.pone.0178481 PMC5453691

[19] Laskus T, Radkowski M, Wang LF, Vargas H, Rakela J. Detection of hepatitis G virus replication sites by using highly strand-specific Tth-based reverse transcriptase PCR. J Virol. 1998;72:3072–5. 9525631 10.1128/jvi.72.4.3072-3075.1998 PMC109756

[20] Smith DB, Basaras M, Frost S, Haydon D, Cuceanu N, Prescott L, et al. Phylogenetic analysis of GBV-C/hepatitis G virus. J Gen Virol. 2000;81:769–80. 10675415 10.1099/0022-1317-81-3-769

[21] Laskus T, Wilkinson J, Gallegos-Orozco JF, Radkowski M, Adair DM, Nowicki M, et al. Analysis of hepatitis C virus quasispecies transmission and evolution in patients infected through blood transfusion. Gastroenterology. 2004;127:764–76. 15362033 10.1053/j.gastro.2004.06.005

[22] Martin M. Cutadapt removes adapter sequences from high-throughput sequencing reads. EMBnet. 2011;17:10–2 2011. 10.14806/ej.17.1.200

[23] Hannon Laboratory. FastX Toolkit. 2015 [cited 2018 Jul 30]. http://hannonlab.cshl.edu/fastx_toolkit/index.html

[24] Larkin MA, Blackshields G, Brown NP, Chenna R, McGettigan PA, McWilliam H, et al. Clustal W and Clustal X version 2.0. Bioinformatics. 2007;23:2947–8. 17846036 10.1093/bioinformatics/btm404

[25] Rozas J, Ferrer-Mata A, Sánchez-DelBarrio JC, Guirao-Rico S, Librado P, Ramos-Onsins SE, et al. DnaSP 6: DNA sequence polymorphism analysis of large data sets. Mol Biol Evol. 2017;34:3299–302. 29029172 10.1093/molbev/msx248

[26] Zuker M. Mfold web server for nucleic acid folding and hybridization prediction. Nucleic Acids Res. 2003;31:3406–15. 12824337 10.1093/nar/gkg595 PMC169194

[27] Jespersen MC, Peters B, Nielsen M, Marcatili P. BepiPred-2.0: improving sequence-based B-cell epitope prediction using conformational epitopes. Nucleic Acids Res. 2017;45(W1):W24–9. 28472356 10.1093/nar/gkx346 PMC5570230

[28] Simons JN, Desai SM, Schultz DE, Lemon SM, Mushahwar IK. Translation initiation in GB viruses A and C: evidence for internal ribosome entry and implications for genome organization. J Virol. 1996;70:6126–35. 8709237 10.1128/jvi.70.9.6126-6135.1996 PMC190635

[29] Bednar MM, Sturdevant CB, Tompkins LA, Arrildt KT, Dukhovlinova E, Kincer LP, et al. Compartmentalization, viral evolution, and viral latency of HIV in the CNS. Curr HIV/AIDS Rep. 2015;12:262–71. 25914150 10.1007/s11904-015-0265-9 PMC4431548

[30] Radkowski M, Wilkinson J, Nowicki M, Adair D, Vargas H, Ingui C, et al. Search for hepatitis C virus negative-strand RNA sequences and analysis of viral sequences in the central nervous system: evidence of replication. J Virol. 2002;76:600–8. 11752151 10.1128/JVI.76.2.600-608.2002 PMC136845

[31] Forton DM, Karayiannis P, Mahmud N, Taylor-Robinson SD, Thomas HC. Identification of unique hepatitis C virus quasispecies in the central nervous system and comparative analysis of internal translational efficiency of brain, liver, and serum variants. J Virol. 2004;78:5170–83. 15113899 10.1128/JVI.78.10.5170-5183.2004 PMC400349

[32] Jørgensen GE, Hammarin AL, Bratt G, Grandien M, Flaegstad T, Johnsen JI. Identification of a unique BK virus variant in the CNS of a patient with AIDS. J Med Virol. 2003;70:14–9. 12629638 10.1002/jmv.10370

[33] Radkowski M, Kubicka J, Kisiel E, Cianciara J, Nowicki M, Rakela J, et al. Detection of active hepatitis C virus and hepatitis G virus/GB virus C replication in bone marrow in human subjects. Blood. 2000;95:3986–9. 10845938

[34] Tucker TJ, Smuts HE, Eedes C, Knobel GD, Eickhaus P, Robson SC, et al. Evidence that the GBV-C/hepatitis G virus is primarily a lymphotropic virus. J Med Virol. 2000;61:52–8. 10745232

[35] Chivero ET, Bhattarai N, Rydze RT, Winters MA, Holodniy M, Stapleton JT. Human pegivirus RNA is found in multiple blood mononuclear cells in vivo and serum-derived viral RNA-containing particles are infectious in vitro. J Gen Virol. 2014;95:1307–19. 24668525 10.1099/vir.0.063016-0 PMC4027039

[36] Chivero ET, Stapleton JT. Tropism of human pegivirus (formerly known as GB virus C/hepatitis G virus) and host immunomodulation: insights into a highly successful viral infection. J Gen Virol. 2015;96:1521–32. 25667328 10.1099/vir.0.000086 PMC5744597

[37] Hickey WF. Leukocyte traffic in the central nervous system: the participants and their roles. Semin Immunol. 1999;11:125–37. 10329499 10.1006/smim.1999.0168

[38] Hickey WF, Vass K, Lassmann H. Bone marrow-derived elements in the central nervous system: an immunohistochemical and ultrastructural survey of rat chimeras. J Neuropathol Exp Neurol. 1992;51:246–56. 1583531 10.1097/00005072-199205000-00002

[39] Unger ER, Sung JH, Manivel JC, Chenggis ML, Blazar BR, Krivit W. Male donor-derived cells in the brains of female sex-mismatched bone marrow transplant recipients: a Y-chromosome specific in situ hybridization study. J Neuropathol Exp Neurol. 1993;52:460–70. 8103085 10.1097/00005072-199309000-00004

[40] Hickey WF, Hsu BL, Kimura H. T-lymphocyte entry into the central nervous system. J Neurosci Res. 1991;28:254–60. 2033653 10.1002/jnr.490280213

[41] Knopf PM, Harling-Berg CJ, Cserr HF, Basu D, Sirulnick EJ, Nolan SC, et al. Antigen-dependent intrathecal antibody synthesis in the normal rat brain: tissue entry and local retention of antigen-specific B cells. J Immunol. 1998;161:692–701. 9670944

[42] Zheng J, Gendelman HE. The HIV-1 associated dementia complex: a metabolic encephalopathy fueled by viral replication in mononuclear phagocytes. Curr Opin Neurol. 1997;10:319–25. 9266156

[43] Davies NW, Brown LJ, Gonde J, Irish D, Robinson RO, Swan AV, et al. Factors influencing PCR detection of viruses in cerebrospinal fluid of patients with suspected CNS infections. J Neurol Neurosurg Psychiatry. 2005;76:82–7. 15608000 10.1136/jnnp.2004.045336 PMC1739313

[44] Radkowski M, Przyjalkowski W, Lipowski D, Wang LF, Laskus T. Lack of GB virus C/hepatitis G virus sequences in cerebrospinal fluid in patients with central nervous system infections. Scand J Infect Dis. 1998;30:539. 10066065 10.1080/00365549850161647

[45] Kaiser R. Tick-borne encephalitis: Clinical findings and prognosis in adults. Wien Med Wochenschr. 2012;162:239–43. 22695809 10.1007/s10354-012-0105-0

[46] Růžek D, Dobler G, Donoso Mantke O. Tick-borne encephalitis: pathogenesis and clinical implications. Travel Med Infect Dis. 2010;8:223–32. 20970725 10.1016/j.tmaid.2010.06.004

[47] Agol VI. The 5′-untranslated region of picornaviral genomes. Adv Virus Res. 1991;40:103–80. 1957717 10.1016/S0065-3527(08)60278-X PMC7130636

[48] Dockter J, Evans CF, Tishon A, Oldstone MB. Competitive selection in vivo by a cell for one variant over another: implications for RNA virus quasispecies in vivo. J Virol. 1996;70:1799–803. 8627703 10.1128/jvi.70.3.1799-1803.1996 PMC190006

[49] McLinden JH, Kaufman TM, Xiang J, Chang Q, Klinzman D, Engel AM, et al. Characterization of an immunodominant antigenic site on GB virus C glycoprotein E2 that is involved in cell binding. J Virol. 2006;80:12131–40. 17035329 10.1128/JVI.01206-06 PMC1676310

[50] Domingo E, Sheldon J, Perales C. Viral quasispecies evolution. Microbiol Mol Biol Rev. 2012;76:159–216. 22688811 10.1128/MMBR.05023-11 PMC3372249

