# Correction: Vol. 24, No. 10

**DOI:** 10.3201/eid2505.C12505

**Published:** 2019-05

**Keywords:** errata, erratum, correction

A grant number was listed incorrectly in Human Pegivirus in Patients with Encephalitis of Unclear Etiology, Poland (I. Bukowska-Ośko et al.). The Polish National Science Center grant number should be 2017/25/B/NZ6/01463. The article has been corrected online (https://wwwnc.cdc.gov/eid/article/24/10/18-0161_article).

