# Supplementary material for: Human Pegivirus in Patients with Encephalitis of Unclear Etiology, Poland
Source: Emerg Infect Dis. 2018 Oct;24(10):1785–94. doi: 10.3201/eid2410.180161 (PMC6154136; doi:10.3201/eid2410.180161)
Supplement: Technical Appendix — Additional information about human pegivirus in patients with encephalitis, Poland. [file 18-0161-Techapp-s1.pdf]

Article DOI: https://doi.org/10.3201/eid2410.180161

Human Pegivirus in Patients with Encephalitis of Unclear Etiology, Poland

Technical Appendix

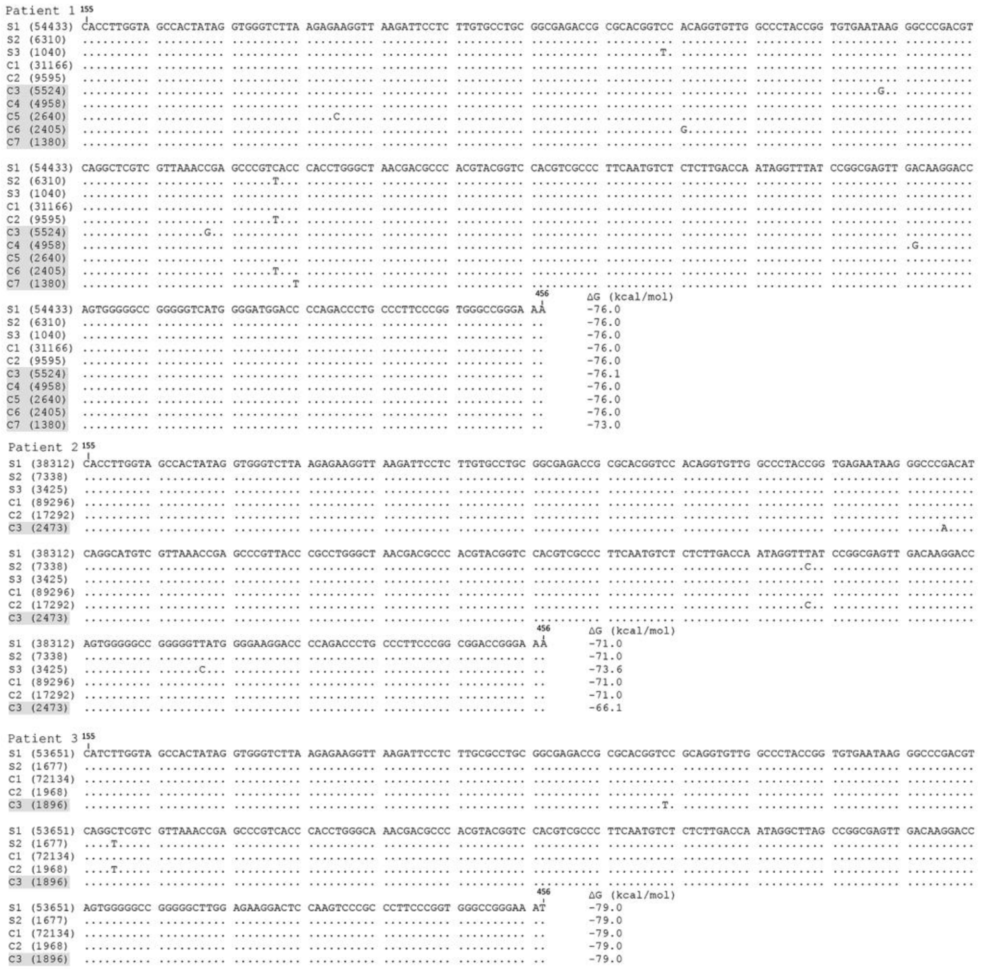

Technical Appendix Figure. Comparison of 5′ untranslated region human pegivirus sequences amplified from serum and cerebrospinal fluid from 3 patients with encephalitis of unclear origin, Poland, 2012–2015. Numbers in parentheses represent the number of reads representing a given sequence. Shading indicates sequences unique to cerebrospinal fluid. Nucleotide numbering follows the reference strain published by Linnen et al (*2*) (GenBank accession no. NC_001710.1). Free energies of the predicted secondary RNA structures were calculated by using Mfold version 3.2 (*26*), and the structures themselves followed the model proposed by Simons et al. (*28*). C, cerebrospinal fluid; S, serum.
